# QAOA for Max-Cut requires hundreds of qubits for quantum speed-up

**DOI:** 10.1038/s41598-019-43176-9

**Published:** 2019-05-06

**Authors:** G. G. Guerreschi, A. Y. Matsuura

**Affiliations:** Intel Labs, Intel Corporation, Santa Clara, CA 95054 USA

**Keywords:** Computational science, Quantum information

## Abstract

Computational quantum technologies are entering a new phase in which noisy intermediate-scale quantum computers are available, but are still too small to benefit from active error correction. Even with a finite coherence budget to invest in quantum information processing, noisy devices with about 50 qubits are expected to experimentally demonstrate quantum supremacy in the next few years. Defined in terms of artificial tasks, current proposals for quantum supremacy, even if successful, will not help to provide solutions to practical problems. Instead, we believe that future users of quantum computers are interested in actual applications and that noisy quantum devices may still provide value by approximately solving hard combinatorial problems via hybrid classical-quantum algorithms. To lower bound the size of quantum computers with practical utility, we perform realistic simulations of the Quantum Approximate Optimization Algorithm and conclude that quantum speedup will not be attainable, at least for a representative combinatorial problem, until several hundreds of qubits are available.

## Introduction

Quantum computers are soon going to satisfy the requirements, both in terms of number and quality of qubits, to perform restricted forms of computation that are beyond what is classically feasible in a reasonable time. This milestone is often called quantum supremacy^1^ and is expected to be achieved in the next couple of years with systems of about 50 qubits. While impressive, these demonstrations will not bring practical advantage to potential users of quantum computers. For example, the random quantum circuits sampling problem that may be used to show quantum supremacy^2,3^ will not help us solve problems of practical interest.

For users interested in solving real-world applications, it is natural to use quantum speedup to quantify the advantage of quantum computers over classical machines. Since quantum algorithms have a computational cost that is either polynomially^4^ or exponentially lower^5^ than their classical counterparts, for larger problem instances quantum algorithms provide an increasing advantage over classical approaches.

However, the quest for quantum speedup has to adapt to the current state of the technology, in which devices have a relatively small number of qubits, all of limited quality. We are entering the NISQ era, the era of Noisy Intermediate Scale Quantum devices^6^. Without active error correction, with a limited coherence budget and with realistic constraints in qubit connectivity, it is questionable whether we can expect any speedup at all and unclear what size of quantum computers one would need to attain speedup.

In advance of mature quantum computing systems able to address these questions experimentally, powerful simulation tools are needed to reproduce the evolution of quantum systems in the presence of noise and to benchmark short-term quantum algorithms. In particular, heuristic algorithms are recently attracting much attention due to their resilience to certain systematic errors. However, it is essential to understand an algorithm’s behavior under realistic hardware errors, as this insight helps us determine which algorithms represent concrete alternatives to classical methods at the current stage of quantum technology.

In this Article we investigate the Quantum Approximate Optimization Algorithm (QAOA)^7^, a hybrid quantum-classical algorithm, and compare its computational cost with a state-of-the-art classical solver (AKMAXSAT^8^) for the NP-hard problem called Max-Cut. The solution of Max-Cut, even if approximate, has practical application in machine scheduling^9^, image recognition^10^ or for the layout of electronic circuits^11^. In our simulations we include both decoherence and dissipation to verify that the quantum algorithm is robust to realistic levels of noise, and consider the compilation of the algorithm in terms of one- and two qubit gates satisfying the hardware connectivity of a square grid with the corresponding routing overhead. Multiple instances at different problem sizes are solved to estimate the absolute time required to run QAOA and extrapolate its scaling cost.

Our findings indicate that solving Max-Cut, a graph partitioning problem, for small graphs with tens of nodes with a quantum computer takes the same time as solving much larger graphs with hundreds of nodes with classical solvers. Based on data from our extensive simulations at small system sizes, and therefore coping with significant extrapolation uncertainty, one can estimate that the performance crossover between the two approaches would require between several hundreds and a few thousands qubits.

## Results

### The Quantum Approximate Optimization Algorithm

The algorithm chosen for this study, namely the Quantum Approximate Optimization Algorithm (QAOA), belongs to the class of hybrid algorithms and requires, in addition to the execution of shallow quantum circuits, a classical optimization process to improve the quantum circuit itself. Since its proposal^7^, QAOA has attracted considerable interest as an application of pre-error-corrected quantum devices^12–17^.

The goal of QAOA is to solve, in an approximate way and by using a shallow quantum circuit, combinatorial problems like constraint-satisfaction (SAT) problems in their “maximization” formulation. In general, SAT problems are characterized by a set of constraints and by the question of whether it is possible to satisfy all constraints at the same time or not. Their “max” form asks what is the maximum number of constraints that can be satisfied at the same time. The latter question is at least as hard as the former one.

What differentiates it from other variational algorithms is that the quantum circuit is determined by the problem instance with very few adjustable parameters, and it is believed that shallow circuits suffice to achieve good approximate solutions. We apply QAOA to solve Max-Cut, a graph partitioning problem defined as follows. Given a graph, assign one of two colors (e.g., black or white) to each vertex. An edge can be “cut” when it connects two vertices of different color. The problem is to assign the colors so that as many edges as possible can be cut at the same time. Max-Cut belongs to the complexity class called NP-hard, and closely related problems have been used as benchmarks in the context of Adiabatic Quantum Optimization and, in particular, with D-Wave Inc. devices^18^. The objective function corresponds to the hermitian operator:

1 $$C=-\frac{1}{2}\sum _{(i,j)\in E}\,{Z}_{i}{Z}_{j}$$

where *E* is the set of edges of the graph to partition, *Z*_*i*_ the Z Pauli matrix of (logical) qubit *i*, and we have neglected an overall constant. The QAOA circuit is obtained by repeating two blocks of quantum operation for a total of *p* times, with *p* being an adjustable parameter. The variational state prepared by the QAOA circuit has the form:

2 $$|\gamma ,\beta \rangle ={e}^{-i{\beta }_{p}B}{e}^{-i{\gamma }_{p}C}\ldots {e}^{-i{\beta }_{1}B}{e}^{-i{\gamma }_{1}C}|+\ldots +\rangle $$

with $B={\sum }_{k}\,{X}_{k}$ being the sum of the X Pauli matrices associated to each vertex of the graph. Notice that the depth of the quantum circuit is approximately linear in the parameter *p*. Expectations are that the number of parameters (given by 2*p*) and circuit depth scale lightly with the problem size.

Notice that the complexity of Max-Cut depends on the regularity and connectivity of the graph itself. Following an existing trend in the community^7,13,14,16^, we consider random 3-regular graphs, defined as having exactly 3 edges per vertex. Extending the analysis to other types of graph is relatively simple, but the numerical results may differ.

### Compiling and scheduling the quantum circuits

The quantum circuit to prepare state $|\gamma ,\beta \rangle$ is intuitively expressed in terms of single qubit rotations for the *B* part and two-qubit phase gates for the part involving *C*. Rotation angles and phases are free to vary in a continuous way in QAOA and their implementation with a finite set of gates (think for example of the universal set {*H*, *T*, *CNOT*}) may be daunting. However, it is easy to envision that the variational parameter is effectively the time-integrated amplitude of the control field acting on the qubits during the rotation: amplifying the signal amplitude corresponds to increasing the rotation angle. Similar considerations are valid for the phase gates, either implemented directly via controlled *ZZ* interactions (tuning the coupling strength or pulse scheme) or in terms of two CNOTs and a rotation (in this case all two-qubit gates are independent of the variational parameters).

Due to limited hardware connectivity, an additional compilation step is necessary to produce executable circuits. A valid schedule must satisfy three constraints: (1) the logical dependencies between the gates forming the algorithm, (2) the exclusive activation of a qubit, which can be involved in at most one gate at a time, and (3) the fact that two-qubit gates can be performed only between connected qubits^19,20^. Here we consider a square grid connectivity and use a novel scheduler approach^20^ that introduces SWAP operations to route the qubits while minimizing the overall circuit depth. For simplicity, we consider that all gates (single-qubit rotations forming the B operator, controlled-phase gates forming C, and SWAP gates for routing) have the same duration. The circuit depth is the relevant metric, even more than the number of gates, when the main source of error is due to decoherence and not to imperfect pulse control. In the Supplementary Information, we provide a full schedule for a typical 8-qubit instance of QAOA with *p* = 4 and show a visual representation of the first few parallel gates.

### Quantum simulations with realistic noise

The simulation of a quantum system is a computationally intensive task, requiring an amount of memory and number of operations that both scale exponentially in the number of qubits. The massively parallel simulator named qHiPSTER (Quantum HIgh Performance Software Testing EnviRonment) has recently been developed to take maximum advantage of multi-processor and multi-node architectures for the simulation of ideal quantum circuits^21,22^. We adapted it by introducing single-qubit decoherence and dissipation using the Noise Gate approach^23^. This method considers that noise acts on each qubit separately (no spatial or time correlations) in between the execution of gates. By unraveling the master equation in terms of a stochastic Schrödinger equation, the authors of^23^ obtained a mathematical equivalence between the density matrix at the end of the circuit and the average over an ensemble of pure states, each evolved via a stochastically “perturbed” quantum circuit. The perturbation comes from including extra noise gates, one after each actual circuit operation, to emulate the effect of noise. In practice, each noise gate corresponds to a single-qubit rotation around a stochastic axis and by a stochastic angle satisfying a suitable Gaussian distribution^21^, and each perturbed circuit is realized by choosing the rotation axis and angle for every noise gate independently.

Reference^24^ describes the first implementation of noise gates in the qHiPSTER simulator, but in that study the circuit was considered composed by sequential gates. Here we extended the implementation by taking into account the parallelism between gates, as expressed in the optimized schedule, with the effect of reducing the number of noise gates to simulate. Notice that the simulation only deals with pure states, but now it requires to be repeated multiple times (one repetition per noise gate realization) for each quantum circuit. Therefore part of the parallelism of qHiPSTER has been devoted to the contemporaneous simulation of the perturbed circuits necessary to achieve convergence: as discussed in the Supplementary Information, we choose to average over 384 noise realizations.

### Computational time cost of QAOA

Since the goal of our work is to understand the potential of quantum hardware, the computational time required by QAOA is not the time to simulate the quantum algorithm, but the absolute time it would have taken to implement it on actual devices. We report the time needed to solve a single instance, obtained by averaging the results of 40 instances at each problem size in the presence of noise.

Despite recent proposals of gradient-based methods, like gradient descent or Quasi-Newton methods, in the context of QAOA^13^ and other variational algorithms^25^, we used the typical choice of the gradient-free Nelder-Mead method^26^ due to the relatively small dimensionality of the parameter space and the high sampling cost of gradient estimation. The Nelder-Mead method is characterized by four parameters corresponding to reflection, expansion, contraction, and shrinkage (or reduction): we used the values 1.1, 1.5, 0.6, and 0.4 respectively.

**Figure 1:**
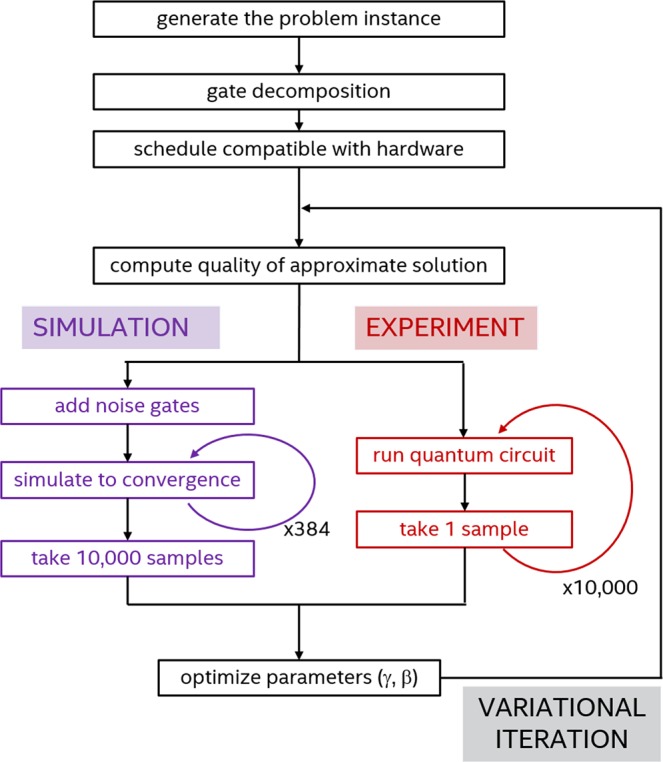
Schematic diagram of the QAOA algorithm and auxiliary tasks to solve a single Max-Cut instance. The two branches, labelled “simulation” and “experiment” respectively, distinguish between the operations to simulate the variational algorithm with classical computers and those to perform it experimentally with quantum devices. In our study, 10000 samples are used to estimate the value of the cost function $\langle \gamma ,\beta |C|\gamma ,\beta \rangle$ at each variational iteration.

Each instance requires several optimization runs that differ due to the randomized initial values of the parameters (*γ*, *β*). This randomization is essential to reach the global solution since each run substantially corresponds to a local optimization, even when the Nelder-Mead simplex is initialized with no locality constraint. Furthermore, each optimization run is composed of hundreds of variational iterations (we limited each run to a maximum 300 updates of the simplex, but the run can end earlier if the best simplex vertex did not change in 10*p* updates) in which the cost function $\langle \gamma ,\beta |C|\gamma ,\beta \rangle$ is estimated by sampling the final state $|\gamma ,\beta \rangle$ in the computational basis. As illustrated in Fig. 1, while this operation can be performed in negligible time during simulations, it requires one experimental repetition of the same experiment for each sample. In practice, one needs (tens of) thousands of repetitions of the same quantum circuit to have enough statistics to estimate the objective function^27,28^.

**Figure 2:**
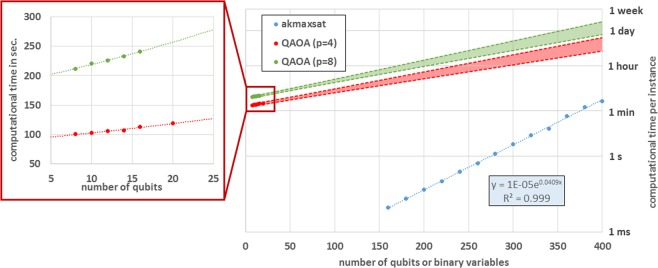
Main panel: Computational cost of solving a single Max-Cut instance on random 3-regular graphs. Blue markers correspond to the classical baseline (AKMAXSAT solver) while red and green marks correspond to the experimental time required by the quantum algorithm QAOA, with *p* = 4 and *p* = 8 respectively. The error bars for the single data points are smaller than the markers (see Supplementary Information). Notice that in the time needed by QAOA to partition graphs with 20 vertices, AKMAXSAT partitions graphs about 20 times larger. The blue dashed line is the result of a fitting procedure with an exponential function. The red and green areas are associated with a 95% confidence interval for the prediction of the QAOA cost based on a linear regression of ${\mathrm{log}}_{{\rm{10}}}(T)$ as a function of the number of qubits (here *T* is the computational time per instance). This extrapolation should be seen as suggesting a qualitative behavior due to the uncertainty in the extrapolation from relatively small system sizes. Insert panel: Magnification of QAOA datapoints. Notice that exponential curves, and smooth curves in general, locally resemble straight lines and this makes it difficult to exclude other functional forms for the extrapolation. It is, however, believed that even quantum computers will not be able to solve NP-hard problems in polynomial time.

The number of experimental repetitions of the coherent quantum computation to solve a single problem instance is therefore given by (20 optimization runs) × (hundreds of function evaluations per run) × (10000 repetitions for statistics). The time duration of a single repetition *T*_s.r._ is given by the number of sequential quantum operations per quantum circuit, and we consider it to be:

$${T}_{{\rm{s}}{\rm{.r}}{\rm{.}}}={T}_{P}+(\mathrm{ciruit}\,\mathrm{depth})\times {T}_{G}+{T}_{M},$$

with *T*_*P*_ being the time to prepare the initial state, *T*_*G*_ the average duration of a quantum gate, and *T*_*M*_ the time needed to measure the qubits. Multiplying *T*_s.r._ by the number of repetitions of coherent computation provides the time cost of a single red or green marker in Fig. 2. The time scales used in our study reflect realistic, but aggressive, projections for state-of-the-art devices based on superconducting circuits^27,29–32^: *T*_*P*_ + *T*_*M*_ = 1 *μ*s, *T*_*G*_ = 10 ns, *T*_2_ = 100 *μ*s, and *T*_1_ = 200 *μ*s.

In Fig. 2 we decided to interpolate the cost of QAOA, obtained from the direct simulation described above, using a functional form that is exponential in the number of qubits. This is counter-intuitive since the duration of a single repetition *T*_s.r._ is linear in *p* and polynomial in the number of qubits, even considering the routing overhead due to the limited connectivity and parallelism. In fact *T*_s.r._ is only part of the cost, the remaining factor being the number of circuits, *i.e*. of (*γ*, *β*) values, that need to be explored during the optimization. The rationale is that the optimization becomes harder for larger systems, possibly due to more local minima^17^ or sharper features of the landscape. This translates into longer optimization runs and possibly, but this is not explored in the current study, the necessity of more independent runs. In the worst case, the number *p* of QAOA steps required for good approximation may also scale exponentially in the number of qubits, even if a recent study seems to indicate that for typical instances of 3-regular MaxCut it is enough for *p* to scale logarithmically^17^. We comment more on this point below, but conservatively fix *p* = 4, 8 in order to avoid an expensive procedure based on trying circuits with variable *p* until convergence which may have a disproportionate impact for small problem sizes.

### Classical alternative

For a meaningful benchmark, the classical baseline must be represented by a state-of-the-art solver that is able to optimize a spectrum of problems similar to the applications of QAOA. A natural choice is considering solvers for constraint-satisfaction problems (called SAT problems) and their “max” formulation.

A good Max-SAT solver that is openly available to the research community, and often utilized in benchmarks of Adiabatic Quantum Optimization devices^33^, is represented by AKMAXSAT^8,34^. It is possible to reduce any Max-Cut problem to a Max-2-SAT problem by a simple reduction: each vertex of the graph corresponds to a distinct binary variable for the SAT, and each edge of the graph gives rise to two constraints (see Supplementary Information for details).

In this case, the computational time is the average time required to solve a single instance with AKMAXSAT running on a single Intel Xeon Phi processor (Performance results are based on testing as of December 20th 2017 and may not reflect all publicly available security updates. See configuration disclosure for details. No product can be absolutely secure. Intel, Xeon, and Intel Xeon Phi are trademarks of Intel Corporation in the United States and other countries. Other names and brands may be claimed as the property of others). The timings reported in Fig. 2 and in the Supplementary Information are averaged over 400 instances of Max-Cut and exploited the parallelism offered by the 68 cores of the Intel Xeon Phi processor in a trivial way: each instance was separately run on a single core, effectively providing a reduction of the total computational time that is strictly linear in the number of cores. No classical strategy can provide more speedup from parallelism than a linear reduction. For consistency, the computational time of a single instance is considered to be equal to the total computational time divided by the number of instances (400 in this case).

### Comparison and performance crossover

The computational cost of both the classical approach and the quantum variational algorithm are showed in Fig. 2, the blue markers corresponding to datapoints for the classical AKMAXSAT, while the red and green markers for QAOA with *p* = 4 and *p* = 8 respectively. We can draw two qualitative conclusions: the first is that, in the time needed to experimentally run QAOA for 20 qubits, AKMAXSAT is able to solve Max-Cut instances corresponding to graphs with about 20 times more nodes. This means that, even in the most favorable scenario, quantum advantage cannot be achieved without hundreds of qubits. Observe also that the noise is relatively weak and corresponds to *T*_2_ = 10^4^ × *T*_*G*_, i.e. 10,000 times the average gate duration (and about 100 times the measurement time *T*_*M*_).

The second conclusion is based on the 95% confidence interval for the predicted exponential behavior of the QAOA cost extrapolated from the small-size datapoints. It appears that, if a crossover between the quantum and classical solvers exists, it takes place for instances with several hundreds of binary variables. While for small-size instances the classical baseline is much faster, beyond the crossover point there would be opportunity for quantum speedup. However, it is important to take into consideration three additional aspects to properly interpret the latter observation.

First, the rigorous extrapolation of the behavior for large problem sizes requires having results for “large enough” instances to avoid being deceived by small-size effects. A priori it is unclear what large enough means in the current context, but it is computationally demanding to perform extensive simulations in the presence of noise beyond 20–24 qubits. The extrapolation is then highly influenced by the choice of the fitting function and possibly unreliable. A close-up of the quantum datasets is presented in the inset panel.

Second, the quantum solver is not guaranteed to reach the best solution, but it only provides a good approximation. This adds a third relevant quantity in addition to problem size and computational time, namely the quality of the approximate solution. We verified that the quantum state produced at the end of the (optimized) QAOA circuit has substantial overlap with the global solution (see Supplementary Information). For 20 qubits and *p* = 4 the overlap is around 10% and the exact solution would most probably be obtained in many of the repetitions required in the protocol. Indeed, the global solution would likely appear much earlier in the optimization process. The extension of our study to classical approximate algorithms would allow the introduction of a third axis in Fig. 2 to characterize and compare the quality of the approximate solutions. Currently the best approximate solvers for Max-Cut are based on the reformulation of the problem in terms of semi-definite programming^35,36^ or following a combinatorial approach^37^.

Third, it is unclear whether *p* = 8 will be sufficient to solve problems involving several hundreds or a few thousands of qubits. Expectations are that *p* should grow, even if relatively mildly, with the number of qubits *N*. While this is not a problem in terms of circuit depth (that would increase only linearly with *p*), we notice that the coefficient in the exponential function increases from 0.0141 for *p* = 4 to 0.0159 for *p* = 8 (the values for the upper bound of the prediction interval are 0.0155 and 0.0173 respectively, slightly larger than those for the extrapolated curves). If this observation is a trend related to increasing *p*, quantum speedup may be compromised if the coefficient overcomes the equivalent one for classical algorithms (0.0409 for AKMAXSAT) before the crossover point is reached.

Finally, a recent study^38^ analyzed QAOA for Max-Cut and reached apparently opposite conclusions supporting “the prospects that QAOA will be an effective method for solving interesting problems on near-term quantum computers”. However the base of the comparison is very different from our study since the resource intensive part of finding the optimal (or at least good) values of the parameters is separated from the solution of the specific instance. Most of the cost of running QAOA is then absorbed into a sort of global training and neglected in the cost-per-instance analysis. While the approach is surely interesting, no specific arguments are provided to quantify the efficacy of global training for larger and larger instances and therefore their claims remain unproven.

## Discussion

In the era of noisy intermediate-scale quantum devices, the first experimental milestone within reach is the so-called Quantum Supremacy: performing certain tasks designed to be unfeasible for current and near-future classical machines. However, potential users of quantum computers would certainly like to gain insight on practical problems either by achieving the solution faster or, in other contexts not explored in this work, by improving the quality of approximate solutions.

We performed extensive simulations of the Quantum Approximate Optimization Algorithm applied to instances of a graph partitioning problem, called Max-Cut, belonging to the NP-hard complexity class. We included all the relevant aspects to make the simulations as realistic as possible. Quantum circuits were decomposed in one- and two-qubit gates and scheduled according to the underlying qubit connectivity^20^, here a bidimensional square grid. Noise was introduced following an approach based on the integration of stochastic Schrödinger equations^23,24^. The statistical uncertainty due to sampling the quantum state at the end of QAOA circuits was taken into account by simulating the very same process (using pseudo-random generators), and it impacted the performance of the classical optimizer.

Our results show that classical solvers are very competitive until several hundreds of variables are considered. Quantum speedup, in terms of the absolute time required and not in scaling, can be achieved only after the performance crossover point marking the break-even condition of quantum and classical methods. We estimate the crossover to be between several hundreds and a few thousands qubits for QAOA applied to the solution of Max-Cut instances for random 3-regular graphs. We emphasize that the exponential cost of the QAOA protocol considered in our study is not due to the quantum circuits being exponentially deep, but to the difficulty of optimizing their variational parameters.

As a final remark, it is interesting to consider how the situation changes by having at our disposal not a single, but a number of quantum devices. Following the protocol described in this work, it is trivial to parallelize the quantum computation with respect to the instance and optimization run. However, the function evaluations encountered in each optimization run must be performed sequentially if one wants to preserve the resilience of hybrid algorithms to systematic errors. In addition, one cannot use multiple quantum devices to accumulate the repetition statistics faster (in fact, each device may be affected by different calibration errors). The parallelism is then limited by the homogeneity of the quantum devices at our disposal and our desire to preserve the robustness of variational algorithms.

## Supplementary information

Supplementary Information: QAOA for Max-Cut requires hundreds of qubits for quantum speed-up

## Data Availability

Data from the numerical simulations can be made available upon request.
