# Supplementary material for: QAOA for Max-Cut requires hundreds of qubits for quantum speed-up
Source: Sci Rep. 2019 May 6;9:6903. doi: 10.1038/s41598-019-43176-9 (PMC6502860; doi:10.1038/s41598-019-43176-9)
Supplement: Supplementary file 1 — Supplementary Information: QAOA for Max-Cut requires hundreds of qubits for quantum speed-up [file 41598_2019_43176_MOESM1_ESM.pdf]

Supplementary Information:
QAOA for Max-Cut requires hundreds of qubits for quantum speed-up

G.G. Guerreschi[1, *] and A.Y. Matsuura[1]

[1] *Intel Labs, Intel Corporation, Santa Clara, CA 95054, USA*

(Dated: December 6, 2018)

I. CONVERGENCE OF SIMULATIONS WITH NOISE

By design, qHiPSTER simulates noiseless quantum circuits [1]. The way one introduces noise, following a mathematically exact approach described in [2, 3], is to add "noise gates" that cause small and stochastic perturbations of the ideal circuit. A single perturbation is not descriptive of the overall noise process, but if the simulation is repeated with many different perturbations, then the average result captures both decoherence and relaxation.

Each perturbation requires an additional noise gate for each actual gate in the quantum algorithm, in this way doubling the total number of operations required by the simulator. However, the main overhead comes from the need to average over the computation of a large number of different perturbations because the mathematical equivalence is only valid in that limit. Our preliminary study indicates that several hundreds of distinct perturbations were necessary to achieve convergence of the quantity involved in the classical optimization, at least for the noise level representative of realistic devices.

FIG. 1 shows how the *estimate* of the approximation ratio changes with the number of noise realizations. Considering a single line, convergence cannot be achieved until the final plateau is reached, whose value corresponds to the asymptotic value for very large number of noise realizations. However this is a necessary but not sufficient condition, since the number of realizations to reach the final plateau may be affected by a particularly lucky or unlucky sequence of the (random) noise gates. One needs to confirm that convergence to the final plateau is reached irrespective of the random number stream. The dashed lines represent different noise realizations of the yellow curve at $T_G/T_2 = 500$ and confirm that the same asymptotic value is reached starting from approximately 400 noise realizations.

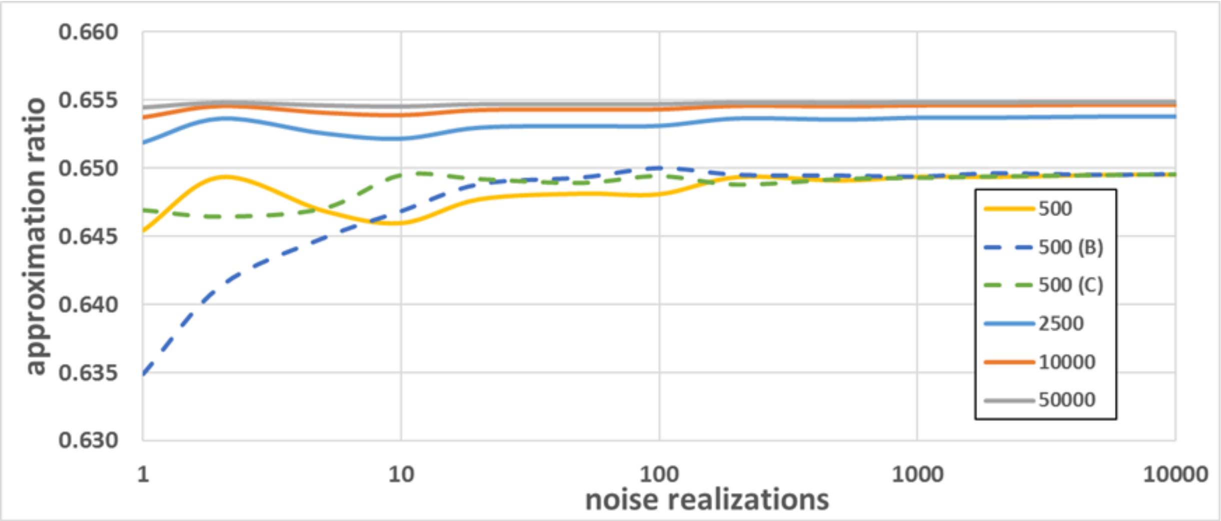

FIG. 1. Example of convergence for noisy simulation of 12-qubit QAOA circuits. A large number of independent perturbations of the ideal quantum circuit are simulated to reproduce the effect of noise. Different curves relates to different noise levels as labelled by the T_2/T_G ratio (with T_2 being the dephasing time, T_G the duration of an average gate, and we considered the relaxation time $T_1 = 2T_2$). The dashed lines represent alternative realizations obtained by initializing the random number generator with different seeds.

* gian.giacomo.guerreschi@intel.com

It is important to specify how the noise level is computed. The relevant quantity is the ratio between the coherence time of the qubits and the duration of a quantum operation. While different operations may take a different amount of time, for simplicity we consider a reasonable value of the average gate duration T_G. We express T_2, i.e. the single qubit dephasing time, as a multiple of the gate duration. For superconducting architectures, aggressive but realistic values are: $T_2 = 100\,\mu$s, $T_1 = 200\,\mu$s and $T_G = 10\,$ns [4–8]. The corresponding curve in FIG. 1 is labelled by "10000", this number representing the ratio T_2/T_G. In our noise model, we assume that the relaxation time T_1 is twice as long as the dephasing time T_2. This happens when the coherence is not limited by relaxation, but effectively describes additional noise sources as a conservative expectation in short term devices.

Finally, let us compare our approach to simulate noise with alternative methods and, in particular, with the density-matrix simulators. There are two main differences: Density-matrix simulators directly describe the evolution of the system without requiring multiple noise realizations, but a single computation is much more costly. In fact, while qHiPSTER requires the storage of 2^N amplitudes and $\mathcal{O}(2^N)$ operations per quantum gate, density-matrices are represented by 2^{2N} complex numbers and require at least $\mathcal{O}(2^{2N})$ for each update. In practice, simulating 20 qubits in presence of noise via density-matrix approaches demands an amount of memory equivalent to simulate 40 qubits with qHiPSTER. Even considering the necessity of a few thousands noise realizations, our approach is more efficient for $N \gtrsim 13$ (conservative estimate since $2^{13} = 8192$).

II. SCHEDULING QUANTUM CIRCUITS ON A SQUARE GRID

Quantum circuits are usually described in terms of one- and two-qubit operations. The latter ones are defined by specifying which logical qubits (not to be confused with encoded qubits since we do not consider active error correction techniques) they involve, without taking into account that such qubits may be associated with physical qubits that are placed in distant parts of the hardware. To allow for a direct interaction, logical qubits must be routed to connected pair of physical qubits, and we use SWAP gates to achieve such task. We apply the framework presented in reference [9] where the output of the compilation is presented in terms of the Physical Data Precedence Table (PDPT): each column corresponds to a physical qubit and each row to a clock-cycle (we assume that all gates are executed in a single clock-cycle), the entry value is an integer that indicates if a qubit is idle (null entry) or if a gate from the algorithm is performed (positive entry, unique for each gate) or if a SWAP gate for routing is performed (negative entry).

Visualizing schedules for bi-dimensional architectures would require a tri-dimensional plot (the extra dimension being time), but one can present snapshot of the schedule for each clock-cycle separately. In Fig. 2 we provide an example of the first three clock-cycles of QAOA with 8 logical qubits on a machine that has $3 \times 3 = 9$ physical qubits. Notice that the first gate of the QAOA algorithm are the two-qubit operations associated with the clauses of the Max-Cut instance, one gate per clause. For this specific instance, the edges of the graph are:

$$E = \big\{(7,6),(7,3),(5,3),(6,2),(6,1),(5,2),(7,4),(3,0),(1,0),(4,1),(5,4),(2,0)\big\}$$

and therefore the first gates of the QAOA circuits are:

$$\begin{aligned} g_1 &= \exp\left(-i\gamma_1 Z_7 Z_6/2\right) \\ g_2 &= \exp\left(-i\gamma_1 Z_7 Z_3/2\right) \\ g_3 &= \exp\left(-i\gamma_1 Z_5 Z_3/2\right) \\ &\vdots \\ g_{12} &= \exp\left(-i\gamma_1 Z_2 Z_0/2\right)\,. \end{aligned}$$

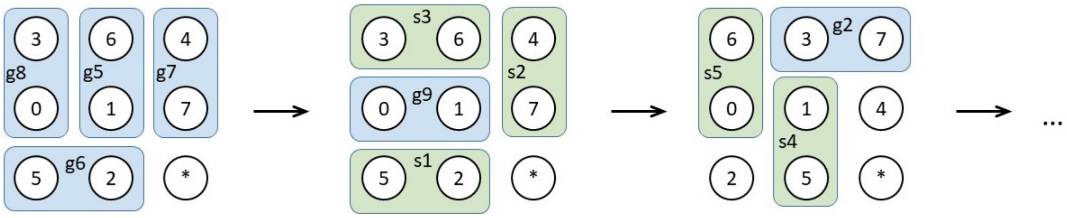

FIG. 2. First three snapshots of the schedule for the QAOA circuit. The logical instance involves 8 qubits and the schedule is compiled for a 3×3 grid of physical qubits, here visualized as circles. Two-qubit gates from the QAOA circuits are depicted in blue, while the SWAP gates for routing are depicted in green. The number inside the circles corresponds to the index of the logical qubit associated with the physical qubit. See text for additional details on the specific Max-Cut instance.

The PDPT file is included for completeness (here the QAOA circuit has $p = 4$).

```
# PDPT: each column is associated to a physical qubit , each row to a clock-cycle
## physical qubit indices ##########################################################
        0       1       2       3       4       5       6       7       8
## logical qubit indices ###########################################################
        3       6       4       0       1       7       5       2       *
####################################################################################
        8       5       7       8       5       7       6       6       0
        -3      -3      -2      9       9       -2      -1      -1      0
        -5      2       2       -5      -4      0       0       -4      0
        0       0       0       4       11      11      4       0       0
        0       3       -8      -6      3       -8      -6      -7      -7
        12      16      0       12      18      -10     -9      -9      -10
        13      23      10      15      23      10      0       1       1
        28      28      17      26      26      14      0       19      20
        0       -11     -12     -13     -11     -12     -13     0       0
        -14     -14     0       0       -15     -15     24      24      0
        -16     29      29      -16     0       22      0       0       22
        -18     -18     0       31      31      36      0       -17     -17
        0       0       -19     38      27      -19     0       27      0
        -20     -21     -21     -20     0       25      0       0       25
        43      43      0       32      30      30      32      21      21
        0       0       0       33      37      34      35      40      39
        -22     0       0       -22     47      45      0       47      45
        48      48      0       46      50      50      46      41      41
        -25     -25     -23     51      51      -23     -24     -24     0
        0       49      49      58      57      0       0       44      44
        0       0       54      71      71      0       0       0       59
        0       -27     0       -28     -27     -26     -28     0       -26
        42      0       65      42      52      65      0       52      0
        56      70      70      60      53      0       0       55      0
        -29     0       0       -29     0       -30     0       0       -30
        67      67      0       68      68      0       0       64      64
        62      77      -31     62      0       -31     66      66      0
        -33     -33     0       63      69      69      63      -32     -32
        0       0       0       76      -34     74      78      -34     0
        0       61      0       0       61      0       0       72      72
        0       80      0       0       79      0       0       73      75
####################################################################################
```

Listing 1. PDPT file with the qubit placement and complete schedule. Each column corresponds to a physical qubit and each row to a clock-cycle: the entry value is an integer that indicates if a qubit is idle (null entry) or if a gate from the algorithm is performed (positive entry, unique for each gate) or if a SWAP gate for routing is performed (negative entry). The first three clock-cycles are visualized in Figure 2.

III. SIMULATION OF MACHINES WITH ADDITIONAL QUBITS

It is realistic to assume that hardware devices may consist of more qubits than those required by the algorithm. Therefore it is important to understand the role of these extra qubits and whether/how they should be included in the simulation.

Consider a quantum circuit involving N qubits scheduled on a machine having $M > N$ physical qubits. For a lineal architecture an optimal schedule will not involve more than N adjacent qubits, and it is intuitive to neglect the remaining $M - N$ qubits when noise has no spatial correlations (i.e. acts on each physical qubit independently).

For a two-dimensional grid, the optimized schedule may involve more than N qubits. While the actual state of the qubit register for the quantum circuit only span N qubits, other qubits may be involved in the routing procedure and, therefore, interact with “logical” qubits through SWAP operations. Notice that in this Section, as for the rest of the document, logical qubit does not refer to an error corrected qubit, but to those qubits forming the logical register of the quantum algorithm.

Let us divide the physical qubit register in two parts: the logical register and the ancilla register. Their composition changes in time due to SWAP operations between qubits belonging to different registers. The total state can be represented as ρ_{L+A}, the observable at the end of the circuit is C_L (since it relates only to the logical register), and

the quantity of interest is computed according to:

$$\begin{aligned}\langle C\rangle &= \mathrm{Tr}_L\left[\mathrm{Tr}_A\left[\rho_{L+A}\right]C_L\right]\\ &= \mathrm{Tr}_{L+A}\left[\rho_{L+A}C_L\otimes\mathbb{I}_A\right]\\ &= \frac{1}{R}\sum_{i=0}^{R-1}\langle\psi^{(i)}_{L+A}|\,C_L\otimes\mathbb{I}_A\,|\psi^{(i)}_{L+A}\rangle\ ,\end{aligned}$$

where the last line describe our method to introduce noise in the simulation by, effectively, representing the mixed state ρ_{L+A} with a set of R pure states $\left\{|\psi^{(i)}_{L+A}\rangle\right\}_{i=0,1,\ldots,R-1}$. Each state $|\psi^{(i)}_{L+A}\rangle$ is generated by a perturbed version of the ideal quantum circuit in which many single-qubit, stochastic gates are added. Since initially the logical and ancilla register are separable, this property is preserved until the end of each perturbed quantum circuit since SWAP gates cannot create entanglement. Then $|\psi^{(i)}_{L+A}\rangle = |\psi^{(i)}_L\rangle\otimes|\psi^{(i)}_A\rangle$ and

$$\begin{aligned}\langle C\rangle &= \frac{1}{R}\sum_i\langle\psi^{(i)}_{L+A}|\,C_L\otimes\mathbb{I}_A\,|\psi^{(i)}_{L+A}\rangle\\ &= \frac{1}{R}\sum_i\langle\psi^{(i)}_L|\,C_L\,|\psi^{(i)}_L\rangle\,\langle\psi^{(i)}_A|\,\mathbb{I}_A\,|\psi^{(i)}_A\rangle\\ &= \frac{1}{R}\sum_i\langle\psi^{(i)}_L|\,C_L\,|\psi^{(i)}_L\rangle\ ,\end{aligned}$$

meaning that the desired quantity can be computed by knowing $|\psi^{(i)}_L\rangle$ without specifying the state of the ancilla register. Therefore, the simulation only needs to include N qubits and not any of the extra qubits. Physically, this is due to the assumptions behind the noise model which does not include spatial or temporal correlations.

IV. PROBABILITY OF OBSERVING THE GLOBAL SOLUTION

Recall the considerations expressed in the section named "Comparison and performance crossover". To access the quality of the solutions provided by QAOA, we report in FIG. IV the overlap between the optimized state $|\gamma,\beta\rangle$ and the exact solution. Notice that even achieving a probability of observing the solution assignment of only 0.1% would imply that, with high probability, at least one of the 10,000 experimental repetitions of the optimized circuit returns the global solution.

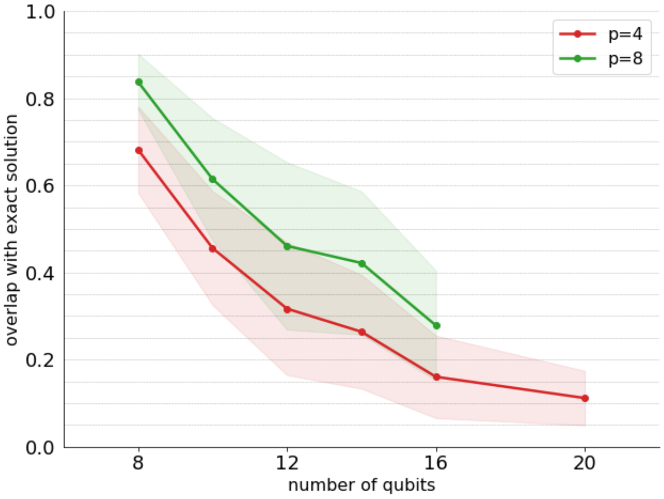

FIG. 3. Overlap with the global solution of the Max-Cut problem for the state at the end of the QAOA circuit after optimization, plotted as a function of the number of qubits or, equivalently, graph vertices. Each point represents the average over 40 instances and the shaded area corresponds to one standard deviation of the distribution.

V. STATISTICS FOR QAOA RESULTS

The results presented in the insert of FIG. 2 (main text)have such small error bars that they are covered by the data markers. In this section, we report the error bar values and describe their significance. First of all, recall the meaning of each datapoint. It represents the absolute time to solve with quantum computers instances of Max-Cut at a certain system size.

For each instance multiple optimization runs are performed, each starting from a different random value of the QAOA parameters, and only the one that achieves the best approximation ratio is associated with the actual (approximate) solution of the instance. However, all runs are required to avoid being deceived by a local maximum. The cost of each optimization run must then be summed up.

Different instances may require a different cost to achieve their solution, even if the number of qubits is the same. This is due to the specific landscape in parameter space that may be more or less smooth and/or present more or fewer local maxima. The time cost to solve a single instance is then the average cost over a set of instances, 40 in our study. From the statistical deviation of the single-instance cost one can associate an error bar to the estimate of the *average* cost according to

$$\text{std. dev. of average} = \sqrt{\frac{\text{variance over instances}}{\text{number of instances}}} \,. \tag{1}$$

Since the standard deviation associated to each point is smaller than the marker in FIG. 2 (main text), the values are provided in Table I below.

N	$p = 4$	$p = 8$
8	100.6 ± 0.7	211.3 ± 0.9
10	102.8 ± 0.7	220.6 ± 1.2
12	106.6 ± 0.6	226.2 ± 1.5
14	107.5 ± 0.9	233.2 ± 1.1
16	113.1 ± 1.0	241.1 ± 1.1
20	118.8 ± 1.0	

TABLE I. Average cost of solving a single instance of Max-Cut with QAOA together with the corresponding standard deviation. The cost corresponds to the absolute time, in seconds, to run the quantum algorithm on realistic hardware. Values correspond to the red and green datapoints in FIG. 2 (main text).

VI. AKMAXSAT RUNNING TIMES

The classical solver used in this study is AKMAXSAT [10], often utilized to benchmark Adiabatic Quantum Optimization devices. For the reduction of Max-Cut to Max-2-SAT we followed the same approach described in reference [11] and that requires a binary variable for each vertex of the graph to be partitioned and two clauses for each edge. Explicitly one has:

$$\begin{aligned} \text{node of graphs: } x_1, x_2 \quad &\rightarrow \quad \text{binary variables: } x_1, x_2\,; \\ \text{edge connecting } x_1 \text{ and} x_2 \quad &\rightarrow \quad \text{clauses: } (x_1 \vee x_2)\,, (\neg x_1 \vee \neg x_2)\,. \end{aligned}$$

It is straightforward to see that at least one clause is always satisfied and that both clauses are satisfied if and only if the edge can be cut, *i.e.* when $x_1 = \neg x_2$. Therefore, if the graph for the Max-Cut problem has E edges, the corresponding Max-SAT instance has $2E$ clauses. If the maximum number of clauses that can be satisfied at the same time is $E + k$ (with $0 < k \leq E$), the solution to the corresponding Max-Cut instance is k.

FIG. 4 provides the computational cost, in absolute time, required by AKMAXSAT to solve one instance (averaged over 400 instances). An exponential fit is provided, together with the regression value $R^2 = 0.9963$. The timings have been obtained using the SSR-NFS computing cluster at PCL (Intel Labs). Jobs were run on a single Intel Xeon Phi 7250 node (68 cores Knights Landing) in parallel, one per core (Performance results are based on testing as of December 20th 2017 and may not reflect all publicly available security updates. See configuration disclosure for details. No product can be absolutely secure. Intel, Xeon, and Intel Xeon Phi are trademarks of Intel Corporation in the United States and other countries. Other names and brands may be claimed as the property of others.).

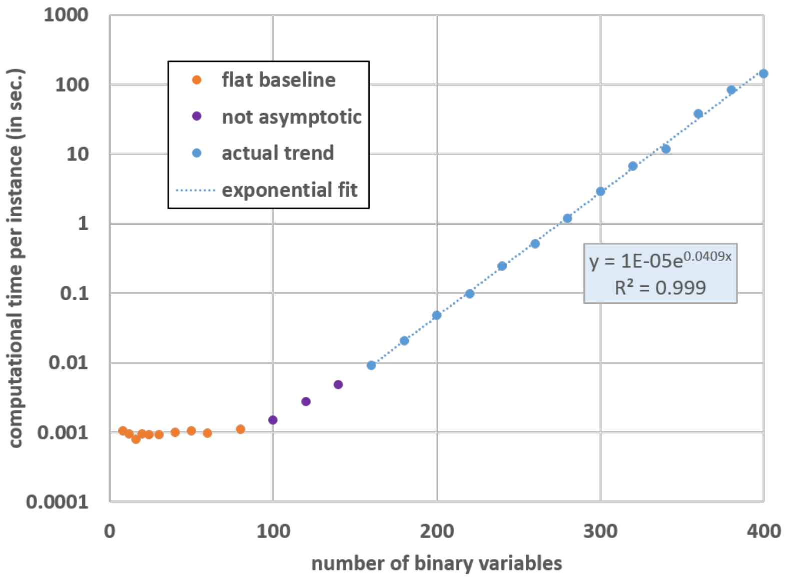

FIG. 4. Computational cost, in absolute time and averaged over 400 instances, to solve a single instance of Max-Cut as a function of the number of nodes in the graph, corresponding to the number of logical qubits for the QAOA algorithm. The classical solver is AKMAXSAT and it was run on a single-socket Intel Xeon Phi 7250 (68 core Knights Landing per socket, turbo enabled, flat, quad, 96 GB).

---

[1] Mikhail Smelyanskiy, Nicolas P. D. Sawaya, and Alán Aspuru-Guzik. qHiPSTER: The Quantum High Performance Software Testing Environment. *arXiv:1601.07195*, 2016.

[2] Angelo Bassi and Dirk André Deckert. Noise gates for decoherent quantum circuits. *Physical Review A*, 77:032323, 2008.

[3] Nicolas P. D. Sawaya, Mikhail Smelyanskiy, Jarrod R. McClean, and Alán Aspuru-Guzik. Error sensitivity to environmental noise in quantum circuits for chemical state preparation. *Journal of Chemical Theory and Computation*, 12(7):3097–3108, 2016.

[4] Michel H. Devoret and Robert J. Schoelkopf. Superconducting circuits for quantum information: An outlook. *Science*, 339(6124):1169–74, mar 2013.

[5] Rami Barends, J. Kelly, A. Megrant, A. Veitia, D. Sank, E. Jeffrey, T. C. White, J. Mutus, Austin G. Fowler, B. Campbell, Y. Chen, Z. Chen, B. Chiaro, A. Dunsworth, Charles Neill, P. J. J. O'Malley, Pedran Roushan, A. Vainsencher, J. Wenner, A. N. Korotkov, A. N. Cleland, and John M. Martinis. Superconducting quantum circuits at the surface code threshold for fault tolerance. *Nature*, 508:500–503, 2014.

[6] P. J. J. O'Malley, Ryan Babbush, Ian D. Kivlichan, Jonathan Romero, Jarrod R. McClean, Rami Barends, J. Kelly, Pedran Roushan, A. Tranter, N. Ding, B. Campbell, Y. Chen, Z. Chen, B. Chiaro, A. Dunsworth, Austin G. Fowler, E. Jeffrey, E. Lucero, A. Megrant, J. Y. Mutus, M. Neeley, Charles Neill, C. Quintana, D. Sank, A. Vainsencher, J. Wenner, T. C. White, P. V. Coveney, Peter J. Love, Hartmut Neven, Alán Aspuru-Guzik, and John M. Martinis. Scalable quantum simulation of molecular energies. *Physical Review X*, 6:031007, 2016.

[7] M Rol, C. C. Bultink, T. E. O'Brien, S. R. de Jong, L. S. Theis, Xiang Fu, F. Luthi, R. F. L. Vermeulen, J. C. de Sterke, A. Bruno, D. Deurloo, R. N. Schouten, F. K. Wilhelm, and Leo DiCarlo. Restless tuneup of high-fidelity qubit gates. *Physical Review Applied*, 7:041001, 2017.

[8] Abhinav Kandala, Antonio Mezzacapo, Kristan Temme, Maika Takita, Jerry M. Chow, and Jay M. Gambetta. Hardware-efficient variational quantum eigensolver for small molecules and quantum magnets. *Nature*, 549(7671):242–246, 2017.

[9] Gian Giacomo Guerreschi and Jongsoo Park. Two-step approach to scheduling quantum circuits. *Quantum Science and Technology*, 3(4):045003, 2018.

[10] Adrian Kügel. Improved exact solver for the weighted Max-SAT problem. *Proc. Pragmatics of SAT Workshop (POS-10)*, 8:15–27, 2012.

[11] Jens Gramm, Edward A. Hirsch, Rolf Niedermeier, and Peter Rossmanith. Worst-case upper bounds for MAX-2-SAT with an application to MAX-CUT. *Discrete Applied Mathematics*, 130:139–155, 2003.
